# Supplementary material for: A Liposomal Formulation Enhances the Anti-Senescence Properties of Nicotinamide Adenine-Dinucleotide (NAD+) in Endothelial Cells and Keratinocytes
Source: Curr Issues Mol Biol. 2025 Sep 5;47(9):722. doi: 10.3390/cimb47090722 (PMC12468054; doi:10.3390/cimb47090722)
Supplement: Supplementary file 1 [file cimb-47-00722-s001.zip › cimb-3728483-supplementary.pdf]

A Liposomal Formulation Enhances the Anti-Senescence Properties of Nicotinamide Adenine-Dinucleotide (NAD+) in Endothelial Cells and Keratinocytes

Supplementary Materials

Supplementary Table S1. Chemical and physical features of the liposomal matrix with and without NAD^+. The liposomal matrix is physically stable at 4 °C with a pH ranging within 6.0–6.5.

		**LIPOSOMAL MATRIX**		**LIPOSOMAL MATRIX +** NAD^+	
	Temperature	**4°C**	**21°C**	**4°C**	**21°C**
**DAY 1**	Look:	homogeneous	**homogeneous**	homogeneous	**homogeneous**
**DATE: 25.09.2018**	Color:	beige, viscous, mildly dull	**beige, viscous, mildly dull**	beige, viscous, mildly dull	**beige, viscous, mildly dull**
	Stability:	ok	**ok**	ok	**ok**
	pH:	6.5	**6.5**	6.5	**6.5**
**DAY 10**	Look:	homogeneous	**homogeneous**	homogeneous	**homogeneous**
**DATE: 04.10.2018**	Color:	beige, viscous, mildly dull	**beige, viscous, mildly dull**	beige, viscous, mildly dull	**beige, viscous, mildly dull**
	Stability:	ok	**ok**	ok	**ok**
	pH:	6.5	**5.4**	6.5	**5.6**
**DAY 30**	Look:	homogeneous	**homogeneous**	homogeneous	**homogeneous**
**DATE: 25.10.2018**	Color:	beige, viscous, mildly dull	**beige, viscous, mildly dull**	beige, viscous, mildly dull	**beige, viscous, mildly dull**
	Stability:	ok	**ok**	ok	**ok**
	pH:	6.0	**4.8**	6.2	**4.9**

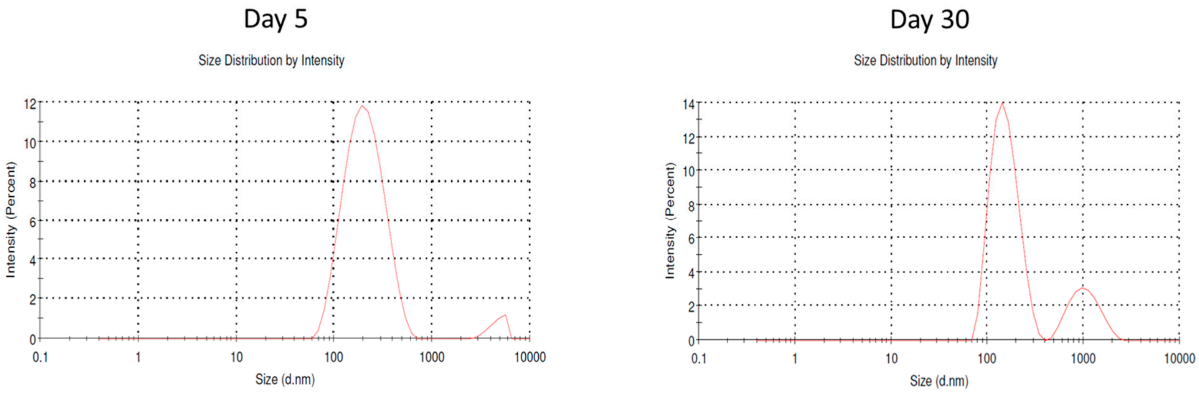

PdI= 0.24; z-average= 192 nm

PdI= 0.25; z-average= 173 nm

Supplementary Figure S1. Size and distribution of the liposomes encapsulating 5% NAD^+, assessed by laser diffraction.

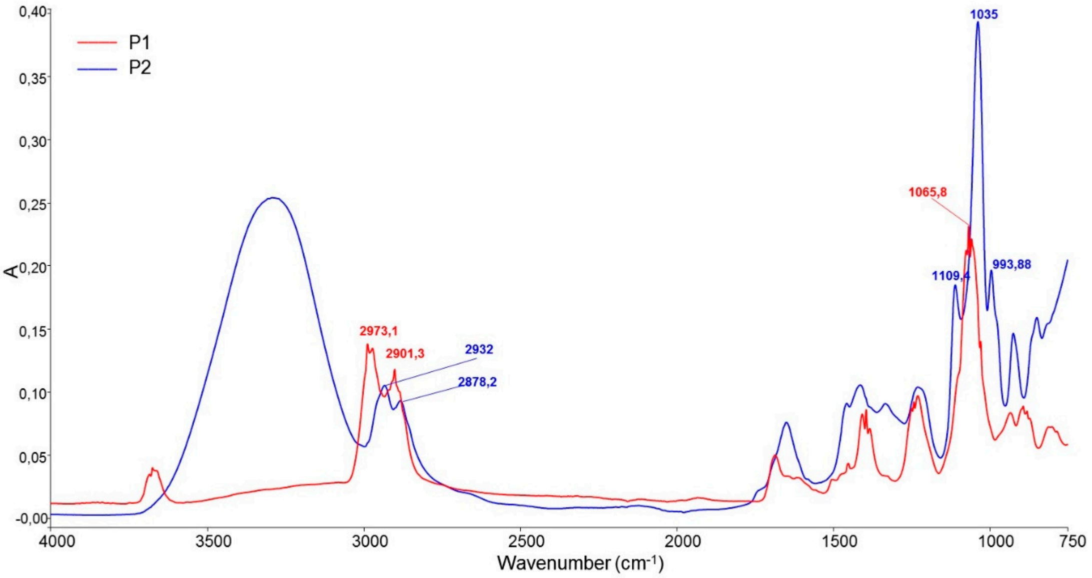

Supplementary Figure S2. Mean infrared absorbed spectra of nicotinamide adenine-dinucleotide (NAD^+) in its powder form (P1, red line) and included in liposomes (P2, blue line).
